# Supplementary material for: Advanced Thermoelectric Performance of SWCNT Films by Mixing Two Types of SWCNTs with Different Structural and Thermoelectric Properties
Source: Materials (Basel). 2025 Jan 4;18(1):188. doi: 10.3390/ma18010188 (PMC11721192; doi:10.3390/ma18010188)
Supplement: Supplementary file 1 [file materials-18-00188-s001.zip › materials-3400368-supplementary.pdf]

Advanced Thermoelectric Performance of SWCNT Films by Mixing Two Types of SWCNTs with Different Structural and Thermoelectric Properties

Yutaro Okano [1], Hisatoshi Yamamoto [1], Koki Hoshino [1], Shugo Miyake [2] and Masayuki Takashiri [1,*]

[1] Department of Materials Science, Tokai University, Hiratsuka 259-1292, Kanagawa, Japan; 3cajm010@mail.u-tokai.ac.jp (Y.O.); 3cajm057@mail.u-tokai.ac.jp (H.Y.); 3cajm049@mail.u-tokai.ac.jp (K.H.)
[2] Department of Mechanical Engineering, Setsunan University, Neyagawa 572-8508, Osaka, Japan; shugo.miyake@setsunan.ac.jp
* Correspondence: takashiri@tokai.ac.jp

Supplementary Information

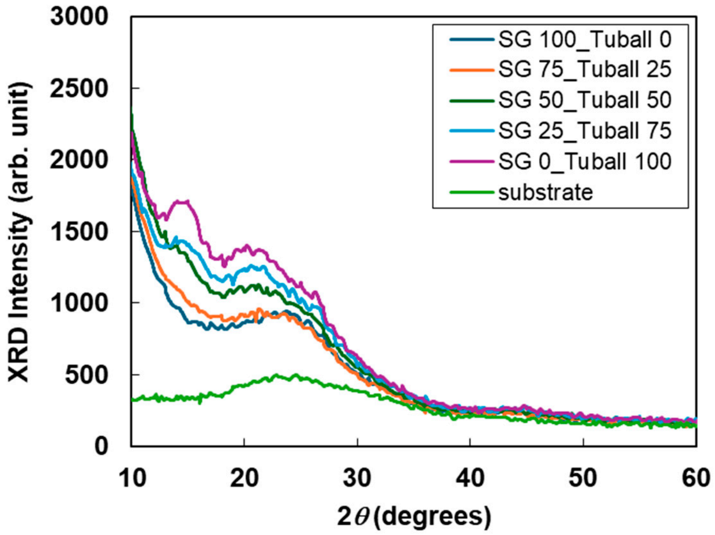

Figure S1: XRD patterns of SWCNT films with different mixing ratios of SWCNTs.

Figure S1 shows the XRD patterns of SWCNT films with different mixing ratios of SWCNTs. The SWCNT film composing solely SWCNT-SG had a broad peak at approximately 2θ=25°. When the ratio of SWCNT-Tu was increased in the SWCNT films, two peaks appeared at lower angles.

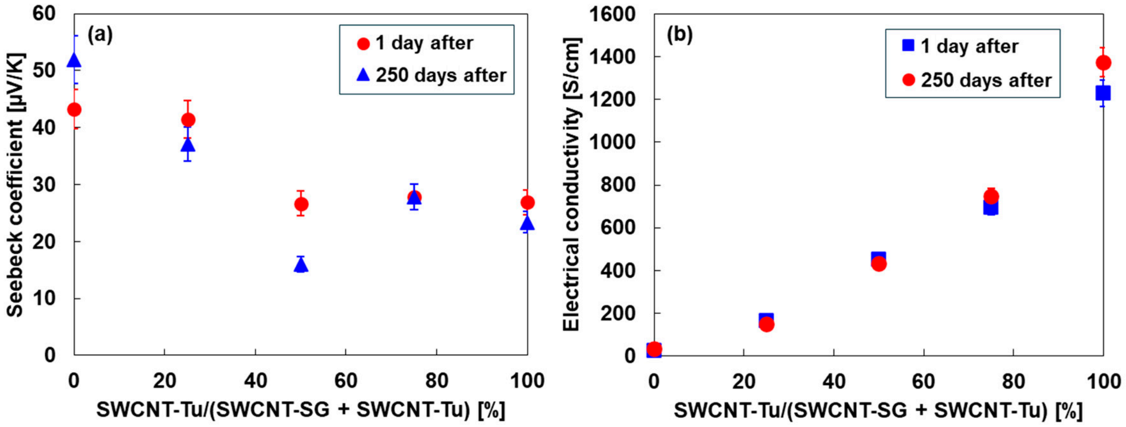

Figure S2: Long-term stability of SWCNT films in the thermoelectric properties with different mixing ratios of SWCNTs. (a) Seebeck coefficient and (b) electrical conductivity.

To investigate the long-term stability of the SWCNT films, we measured their thermoelectric properties again 250 days after the film preparation, as shown in Fig. S2. No significant deteriorations in the Seebeck coefficient (Fig. S2(a)) and the electrical conductivity (Fig. S2(b)) were observed even 250 days after the film was fabricated. Therefore, we conclude that SWCNT films in this study have long-term stability.

Table S1: Comparison of ZT values of SWCNT films and other thermoelectric materials.

Thermoelectric materials	*p*- or *n*-type	*ZT*	References
SWCNT films	*p*-type	1.1×10^{-3} (300 K)	This study
CNT/Bi_2Te_3 composite	*n*-type	0.85 at 473 K	[55]
FeNbSb based half-Heusler	*p*-type	1.1 at 1100 K	[56]
$Cu_3Sb_{0.975}Sn_{0.025}Se_4$	*p*-type	0.75 at 673 K	[57]
$Bi_{0.3}Sb_{1.7}Te_3$	*p*-type	1.3 at 380 K	[58]
PbSeTe/PbTe superlattice	*n*-type	1.1 at 300 K	[59]
(Zr,Hf)Co(Sb,Sn) half-Heusler	*p*-type	0.5 at 1000 K	[60]
